# Mediators of energy homeostasis in hyperthyroidism

**DOI:** 10.20945/2359-3997000000511

**Published:** 2022-08-04

**Authors:** Avinash Patil, Suresh Vaikkakara, Mani Deepthi Dasari, Sandeep Ganta, Alok Sachan, Kiranmayi S. Vinapamula

**Affiliations:** 1 Karnataka Institute of Medical Sciences Department of Medicine Huballi India Department of Medicine, Karnataka Institute of Medical Sciences, Huballi, India; 2 All India Institute of Medical Sciences Department of Endocrinology and Metabolism Mangalagiri India Department of Endocrinology and Metabolism, All India Institute of Medical Sciences, Mangalagiri, India; 3 Chinmaya Mission Hospital Department of Endocrinology Bangalore India Department of Endocrinology, Chinmaya Mission Hospital, Bangalore, India; 4 Kamineni Hospital Department of Endocrinology Hyderabad India Department of Endocrinology, Kamineni Hospital, Hyderabad, India; 5 Sri Venkateswara Institute of Medical Sciences Department of Endocrinology Tirupati India Department of Endocrinology, Sri Venkateswara Institute of Medical Sciences, Tirupati, India; 6 Sri Venkateswara Institute of Medical Sciences Department of Biochemistry Tirupati India Department of Biochemistry, Sri Venkateswara Institute of Medical Sciences, Tirupati, India

**Keywords:** Thyrotoxicosis, body composition, fibroblast growth factor 21, leptin, ghrelin, adiponectin, insulin resistance

## Abstract

**Objective:**

The aim of this study was to assess the effect of hyperthyroidism and its treatment on body weight and composition, insulin resistance, and mediators of appetite and energy homeostasis, namely ghrelin, leptin, adiponectin, and fibroblast growth factor 21 (FGF21).

**Subjects and methods:**

Thirty-five adult patients (27 female and 8 male, aged 39.63 ± 9.70 years) with overt hyperthyroidism were evaluated for leptin, ghrelin, adiponectin, and FGF21 levels; insulin resistance; and body composition using DEXA both at baseline and a minimum of two months following normalization of serum thyroxin on carbimazole treatment. Comparison of means between the baseline and post treatment values was performed by the paired t test for normally distributed parameters and by the Wilcoxon signed-rank test for non-normally distributed data.

**Results:**

Hyperthyroidism correction resulted in an increase in weight from 51.15 ± 8.50 kg to 55.74 ± 8.74 kg (P < 0.001), paradoxically accompanied by a decrease in insulin resistance as measured by HOMA-IR from 1.35 (1.02-1.72) to 0.73 (0.52-0.93) ( *P* < 0.001). Correction of hyperthyroidism was also associated with a decrease in FGF21 from 58 (55-64) to 52 (47-58) pg/mL ( *P* < 0.001) and in leptin levels from 17 (7-36) to 11 (4.6-28) ng/mL ( *P* = 0.03).

**Conclusion:**

Despite lower body weight, thyrotoxicosis is associated with insulin resistance. High levels of thermogenic hormones, leptin, and FGF21 were observed in thyrotoxicosis and may be partly responsible for the excessive heat production typical of this condition.

## INTRODUCTION

Thyrotoxicosis is associated with increased energy production and weight loss despite the presence of an increased appetite ( 1 , 2 ). Thus, thyrotoxicosis is a useful model for understanding the role of the thyroid hormones in regulating weight, energy production, and appetite, either directly or through various mediators like leptin, ghrelin, and fibroblast growth factor 21 (FGF21).

There are few but often conflicting reports about the levels of these hormone mediators in thyrotoxicosis. FGF21, which promotes thermogenesis, has been reported to be elevated in thyrotoxicosis ( 3 , 4 ). Leptin, which is an anorexigenic hormone released from adipose tissue, has been variously reported to be normal ( 5 ), high ( 6 ), or even low ( 7 , 8 ) in different studies in patients of hyperthyroidism. Adiponectin in hyperthyroidism has been observed to be comparable to that in euthyroid controls ( 6 , 8 ). Despite the increase in appetite, serum levels of the “hunger hormone” ghrelin in thyrotoxicosis have been reported to be normal ( 9 , 10 ) or low ( 11 , 12 ).

There have been no studies reporting changes in all of the above hormones influencing weight, appetite, and energy production concomitantly after treatment of thyrotoxicosis in a longitudinal before-after manner, nor have the changes in the levels of these hormones been assessed in the context of changes in body composition during the course of treatment for hyperthyroidism.

The aim of this study was to assess the effect of hyperthyroidism and its treatment on body weight and composition, insulin resistance, and mediators of appetite and energy homeostasis, namely ghrelin, leptin, adiponectin, and FGF21. The primary research questions in this study are a) whether there is any alteration in peripheral signals that control appetite, namely leptin and ghrelin, in thyrotoxicosis, b) whether levels of the thermogenic hormones FGF21 and leptin are altered in thyrotoxicosis, and c) what the influence of excess thyroid hormones on weight, body composition, insulin resistance, and adipokines is.

## SUBJECTS AND METHODS

This was a longitudinal before-after interventional study. The project was approved by the institutional ethics committee of the Sri Venkateshwara Institute of Medical Sciences, Tirupati, with IEC no-780 dated 03/07/2018.

Patients of either sex attending Outpatient Services at the Department of Endocrinology of the Sri Venkateswara Institute of Medical Sciences, Tirupati with newly diagnosed (previously untreated) or inadequately treated primary hyperthyroidism, aged more than eighteen years, who had a serum Free T4 > 1.6 ng/dL and TSH < 0.1 mIU/L were recruited after obtaining their informed consent. Patients with known or newly discovered diabetes mellitus, chronic kidney disease, liver or cardiac disease, or any other chronic illness; those receiving medications known to alter glucose, lipid, and weight homeostasis (e.g., glucocorticoids, metformin etc.); and pregnant women were excluded from the study. The patients’ height was recorded on a stadiometer. Blood samples of the enrolled patients were obtained between 8 am and 9 am after an overnight fast to test for plasma glucose (FPG), free thyroxine (free T4), thyroid stimulating hormone (TSH), and insulin. Insulin resistance was calculated from fasting glucose and insulin using the homeostatic model assessment (HOMA-IR) ( 13 ) Additional aliquots were stored at -40 °C for measurement of FGF21, leptin, ghrelin, and adiponectin.

Free T4, TSH, and insulin were measured on the Access II automated chemiluminescence immunoassay system by Beckman Coulter Inc, USA. The minimum and maximum detection limits for the Free T4 assay were 0.25 and 6 ng/mL, respectively, and those for TSH were 0.0003 and 100 μIU/mL, respectively. The range of detection for insulin was 0.03 to 300 μIU/mL. Leptin and adiponectin were estimated by enzyme-linked immunosorbent assay (ELISA) using kits from Diagnostics Biochem Canada Inc, Ontario, Canada. The detection range for leptin was 0.5-100 ng/mL, and that for adiponectin was 0.06 to 50 ng/mL. Plasma acylated ghrelin was measured by ELISA (Human GHRL, Biocodon Technologies, Kansas, USA) The lower detection limit of the assay was 0.01 ng/mL, and the upper detection limit was 10 ng/mL. Human FGF21 was also measured by ELISA (Human GHRL, Biocodon Technologies, Kansas, USA). The lower detection limit of the assay was 2.49 pg/mL, and the upper detection limit was reported as 1,500 pg/mL. Glucose levels were measured by the glucose oxidase-peroxidase method using a Beckman Olympus AU680 auto analyzer.

Whole body composition was then analyzed with a dual-energy x-ray absorptiometer (Discovery DXA System, Hologic Inc, Massachusetts, USA). Patients were told not to take calcium or multivitamin supplements and antacids for 24 hours before their DEXA scan. They were advised to remove metal objects such as keys, belt, etc. and to wear hospital gowns. Premenopausal women were asked about their last menstrual periods, and a urine pregnancy test was ordered to rule out pregnancy when the menstrual periods were delayed. The following parameters were noted in kg: total body mass, fat mass, lean body mass, bone mineral content (BMC), and lean body mass + bone mineral content, both for the body as a whole and for different regions, namely: both arms, both legs, trunk, head, and the android and gynoid regions (as specified by the Hologic software, reflecting the fat around the waist and hip, respectively). Additionally, certain adipose indices, namely, the percentage of fat in the whole body (%), the trunk/limb fat mass ratio, the percentage of fat in the trunk/percentage of fat in the legs ratio, the fat mass/(height)^2^ ratio (kg/m^2^), and the Android/Gynoid ratio, were recorded. Total body mass estimated on the DEXA scan was used to calculate the body mass index (BMI).

Patients were then started on carbimazole (30 to 45 mg/day) and were periodically followed up with regular estimation of free T4 till it was within the normal range (0.5-1.6 ng/dL). Once the patient had been restored to the euthyroid state, the dose of carbimazole was reduced as required (based on frequent monitoring of free T4) to maintain a near euthyroid state for a further two months. After two months or more of documentation of near euthyroidism, the following investigations were repeated as before: serum free T4, TSH, fasting plasma glucose, insulin, FGF21, ghrelin, leptin, and adiponectin. Whole body composition analysis by DEXA was also repeated.

### Statistical analysis

Continuous variables with normal distribution were expressed as mean ± SD, while those not having normal distribution were expressed as the median (IQR). Correlation between various anthropometric and body composition parameters at baseline was assessed by determining the Spearman’s rank correlation coefficient. Comparison of means between baseline and posttreatment values was performed using the paired *t* test for normally distributed parameters. For non-normally distributed data, the Wilcoxon signed-rank test for paired data was used to compare the median (IQR) between pre- and posttreatment points of time. *P* < 0.05 was considered as significant for all the above tests. Statistical analysis was carried out using SPSS statistical software version 25.

## RESULTS

A total of 35 patients completed the study, of whom 27 were female and 8 were male.

The mean age of the 35 patients who completed the study was 39.63 ± 9.7 years. The mean free T4 of the patients at the time of study entry was 3.73 ± 0.98 ng/dL. All patients had suppressed levels of TSH (<0.1 mIU/L).

### Correlation between anthropometric and body composition parameters vs biochemical parameters at recruitment

Among baseline anthropometric and body composition parameters recorded (as mentioned above in methods), a positive correlation of leptin with the body mass index, total fat mass, the percentage of fat in the whole body and the fat mass/height^2^ ratio was observed, while there was an inverse correlation both with lean body mass and the sum of lean body mass and bone mineral content (see Table 1 ). Adiponectin had significant negative correlation with the android/gynoid fat ratio, the percentage of fat in trunk/percentage of fat in legs ratio and the trunk/limb fat mass ratio, while it did not correlate with any other parameters of body composition. FGF21 also correlated negatively with trunk/limb fat mass ratio at the baseline. No other significant correlations with anthropometric or body composition parameters were observed for these three hormones.

**Table 1:** Correlations (by Spearman’s rank correlation coefficient) observed between baseline hormones and various anthropometric and body composition parameters *

Hormone	Anthropometric/body composition parameter	Spearman’s rho (ρ)	*P* value
Leptin	Body mass index	0.45	0.01
Leptin	Lean body mass	-0.34	0.04
Leptin	Lean body mass + bone mineral content	-0.36	0.03
Leptin	Total fat mass	0.57	<0.01
Leptin	Percentage of fat in whole body (%)	0.66	<0.01
Leptin	Fat mass/height^2^	0.61	<0.01
Adiponectin	Android/gynoid fat ratio	-0.35	0.032
Adiponectin	Percentage of fat in trunk/percentage of fat in legs ratio	-0.39	0.01
Adiponectin	trunk/limb fat mass ratio	-0.35	0.03
FGF -21	trunk/limb fat mass ratio	-0.355	0.03

* Only significant correlations are shown.

Ghrelin and free T4 did not correlate with any of the anthropometric or whole-body composition parameters.

Leptin was positively correlated with insulin (ρ = 0.55; *P* = 0.001) and the HOMA-IR (ρ = 0.49; *P* = 0.003). There were no significant correlations of adiponectin, FGF21, or ghrelin individually with any of the other biochemical parameters, namely, insulin, fasting plasma glucose, HOMA-IR, and free T4. Also, there were no significant correlations of free T4 with insulin, glucose, or HOMA-IR.

### Effect of correction of hyperthyroidism on weight and body composition

Achievement of a euthyroid state was accompanied by a significant gain in weight. Other parameters such as total body fat, lean mass, fat-free mass, and total mass also showed a significant increase after treatment. However, there was no change in the percentage of body fat (see Table 2 ). Treatment of hyperthyroidism resulted in increased calculated adipose indices such as fat mass/height^2^ ratio as well as increases in some of the indices that signify truncal adiposity such as percentage of fat in trunk/percentage of fat in legs ratio and the trunk/limb fat mass ratio, although the android/gynoid ratio did not change ( Table 2 ).

**Table 2:** Body composition parameters before and after treatment

Parameter	Before	After	*P* value (by paired t test)
Total body mass (kg)	51.15 ± 8.50	55.74 ± 8.78	**<0.001**
BMI (kg/m^2^)	21.23 ± 3.5	23.1 ± 3.6	**<0.001**
BMC (kg)	1.94 ± 0.49	1.87 ± 0.32	0.35
Fat mass (kg)	18.69 ± 6.14	20.45 ± 6.49	**<0.001**
Lean body mass (kg)	30.60 ± 5.28	33.36 ± 5.77	**<0.001**
Lean body mass + BMC (kg)	32.41 ± 5.52	35.28 ± 6.10	**<0.001**
Percentage of fat in whole body (%)	36.14 ± 7.61	36.38 ± 7.55	0.512
Fat mass/height^2^ (kg/m^2^)	7.86 ± 2.82	8.60 ± 3.03	<0.001
Android/gynoid ratio	0.86 ± 0.19	0.89 ± 0.15	0.387
Percentage of fat in trunk/percentage of fat in legs ratio	0.74 ± 0.10	0.80 ± 0.11	<0.001
Trunk/limb fat mass ratio	0.76 ± 0.15	0.81 ± 0.14	<0.001

### Changes in biochemical parameters after the correction of hyperthyroidism

After the achievement of euthyroidism, while free T4 became normal, FPG, insulin, and HOMA-IR fell significantly. While FGF21 and leptin showed a decrease, ghrelin showed an increase with treatment of the hyperthyroidism. There was no change noted in adiponectin levels ( Table 3 ).

**Table 3:** Change in biochemical parameters before and after treatment

	Before	After	*P* value
Free T4 (ng/dL)	3.73 ± 0.98	0.8 ± 0.2	**<0.001**
FPG (mg/dL)	94.23 ± 8.75	85.6 ± 6.04	**<0.001**
Insulin * (μIU/mL)	0.8 (0.63-1.07)	0.47 (0.35-0.61)	**<0.001**
HOMA-IR *	1.35 (1.02-1.72)	0.73 (0.52-0.93)	**<0.001**
FGF21 * (pg/mL)	58 (55-64)	52 (47-58)	**<0.001**
Ghrelin * (ng/mL)	0.48 (0.44-0.50)	0.50 (0.44-0.50)	**0.05**
Leptin * (ng/mL)	17 (7-36)	11 (4.6-28)	**0.03**
Adiponectin * (ng/mL)	9 (6-12.5)	8.4 (6-12.5)	0.982

Free T4: free thyroxine; FPG: fasting plasma glucose; HOMA-IR: insulin resistance assessment by homeostatic model assessment; FGF21: fibroblast growth factor 21.

* Data not normally distributed, hence summarized as the median (25^th^-75^th^ centile); the test used to obtain the *P* value was the Wilcoxon signed rank test. The rest of the data is normally distributed and therefore means were compared by a *t* test.

## DISCUSSION

### Hyperthyroidism: effect on weight and body composition

Increased calorigenesis ( 1 , 2 ) leads to weight loss in the hyperthyroid state. This appears to include the loss of both lean body mass and total body fat, whereas the opposite occurs with the correction of hyperthyroidism. We observed an increase in body weight, fat mass, and lean body mass after the correction of hypothyroidism. Although there was no increase in the percentage of fat in the whole body after treatment, there seemed to be a redistribution of fat towards the trunk, with an increase in the trunk/limb fat mass ratio as well as in the percentage of fat in trunk/percentage of fat in legs ratio. Thus, weight gain following treatment of hyperthyroidism is associated with gain in both fat and lean body mass, along with a redistribution of fat towards the trunk. A similar gain in both weight and lean body mass, though without any change in the fat mass, was also reported earlier ( 6 ).

### Hyperthyroidism and insulin resistance

Normally, both weight gain and redistribution of fat to the truncal region should be associated with an increase in insulin resistance. But the treatment of hyperthyroidism both in this study and in previous studies ( 6 ) was observed to be associated with a decrease in glucose, insulin, and insulin resistance as measured by HOMA-IR. Thus, the thyroid hormone itself may be the major contributor to insulin resistance in thyrotoxicosis. As shown in the present study, treatment of hyperthyroidism is thus associated with the added clinically relevant benefit of a reduction in glucose through a decrease in insulin resistance. This may be of benefit to patients with prediabetes or diabetes who also have thyrotoxicosis.

Thyroxine stimulates lipolysis and gluconeogenesis ( 14 ) and is associated with increased sympathetic activation, as evidenced by clinical features like tachycardia, stare, tremor, muscle weakness, and occasional hypokalemic periodic paralysis, which are relieved by the use of non-selective β adrenergic receptor blockers like propranolol. These metabolic effects may be responsible for insulin resistance in hyperthyroidism despite weight and fat loss.

### Hyperthyroidism and FGF21

FGF21 at baseline correlated negatively with trunk/limb fat mass ratio ( *P* = 0.03). Since the truncal fat mass is a storage depot for calories, increased truncal adiposity is evidence of nutritional thrift. FGF21, on the other hand, promotes energy expenditure ( 15 ) rather than storage. It is thus not surprising that it has a negative correlation with the trunk/limb fat mass ratio.

We found that serum FGF21 levels fell after the treatment of hyperthyroidism. In two other studies ( 3 , 4 ), FGF21 was higher in patients with thyrotoxicosis than in healthy euthyroid controls and fell with treatment. Thus, there is a consistent pattern suggesting that FGF21 is elevated in hyperthyroidism, with reduction after treatment.

There is increased calorigenesis ( 1 , 2 ) in hyperthyroidism which is said to be in part due to uncoupling of oxidation from phosphorylation, the uncaptured energy being dissipated as heat. It is known that thyroxine upregulates uncoupling protein 1 (UCP-1) in brown adipose tissue (BAT) ( 16 ). UCP-1 abolishes the proton gradient between the inner and outer aspects of the mitochondrial membrane, thereby preventing energy capture as adenosine triphosphate. FGF21 is also known to upregulate UCP-1( 17 ) in the process of converting white adipose tissue to beige adipose tissue following exposure to a cold environment. These same mechanisms might be operating in the hyperthyroid state, with elevated thyroid hormone levels being associated with increased FGF21 levels and both leading to increased calorigenesis through upregulation of UCP-1 or through other unknown mechanisms. Since circulating FGF21 is most likely of hepatic origin ( 18 ), there may be a direct effect of thyroxine on the liver causing increased production of FGF21.

### Hyperthyroidism and adipocytokines

There is an inverse relationship between adiponectin and body mass, particularly fat mass. Thus, obese persons have lower adiponectin than their lean counterparts ( 19 ). In this study, it was observed that at baseline, there was a negative correlation of adiponectin with the android/gynoid fat ratio, the percentage of fat in trunk/percentage of fat in legs ratio, and the trunk/limb fat mass ratio. Thus, even in thyrotoxicosis, the inverse relation between adiponectin and truncal adiposity ( 19 ) remains intact.

Among the adipocytokines, adiponectin is known to have an insulin-sensitizing role ( 19 ). However, in our study, there was no correlation between serum insulin or HOMA-IR levels and plasma adiponectin concentrations, nor there was any change in adiponectin levels after treatment, despite a significant reduction in insulin resistance. Other studies ( 6 , 8 ) also showed comparable adiponectin levels between hyperthyroid patients and euthyroid controls and no changes in adiponectin levels following treatment of hyperthyroidism. These observations suggest that insulin resistance associated with hyperthyroidism is not related to changes in plasma adiponectin levels.

Leptin, the product of the *ob* gene, is produced mainly in the adipose tissue. It suppresses appetite and increases energy expenditure, thereby lowering body weight. Leptin levels are proportional to adipose tissue mass, i.e., high in obese patients and low in patients with low body fat. Leptin is thus a signal of body fat stores ( 20 ). Even in hyperthyroid patients, we observed a positive correlation of leptin with BMI, total fat mass, fat mass/height ( 2 ), and percentage of fat in the whole body. There was also a positive correlation with plasma insulin and HOMA-IR.

Hyperthyroidism was associated with lower body fat, but, intriguingly, leptin levels were actually found to be higher in the thyrotoxicosis state. Counterintuitively, the levels of leptin fell after treatment of thyrotoxicosis, despite the gain in fat mass. Higher serum leptin levels in thyrotoxic patients compared to age- and BMI-matched euthyroid controls have been reported in another study ( 6 ). It also reported a decrease in leptin following treatment of thyrotoxicosis, as observed in this study. In another study in patients with thyrotoxicosis ( 5 ), the ratio of observed leptin levels to expected leptin levels (based on BMI), expressed as a percentage, fell after treatment.

This would suggest that thyroxine directly increases leptin production in adipose tissue. Indeed, Yoshida and cols. ( 21 ) showed that tri-iodo thyronine enhances leptin mRNA expression in isolated 3T3-L1 adipocytes in cell culture. As leptin enhances energy expenditure ( 20 ), it is possible that high leptin in thyrotoxicosis may contribute to the increased energy production in thyrotoxicosis. In promoting energy expenditure, it may function in conjunction with FGF21.

### Hyperthyroidism and ghrelin

Ghrelin promotes food intake through activation of neurons releasing orexigenic neuropeptides like neuropeptide Y/agouti-related protein and orexin ( 22 ). Though hyperthyroidism is characterized by an increased appetite, this could not be attributed to increased ghrelin in thyrotoxicosis as we rather observed a rise in ghrelin levels with the treatment of thyrotoxicosis ( *P* = 0.05). Two other studies ( 11 , 12 ) showed reduced ghrelin in comparison to euthyroid controls in patients with thyrotoxicosis. In both studies the levels of ghrelin normalized after treatment of the thyrotoxicosis.

It is indeed quite a paradox that, while the anorexigenic hormone leptin is elevated in thyrotoxicosis and its orexigenic counterpart ghrelin is reduced, appetite is increased in thyrotoxicosis. These findings suggest resistance to the anorexigenic action of leptin in thyrotoxicosis. There is a need to investigate central mechanisms of appetite control in thyrotoxicosis, such as the activity of specific classes of appetite-regulating neurons in the hypothalamus.

To conclude, thyrotoxicosis is an insulin-resistant state, despite lower weight and reduced whole body fat mass. Reduction in insulin resistance may be an additional clinically useful outcome of the treatment of thyrotoxicosis. As both FGF21 and leptin are elevated in thyrotoxicosis and as both are known to enhance energy expenditure, they may play an additive or synergistic role in promoting heat production in this condition. Paradoxically, despite the widely reported increased appetite in thyrotoxicosis, in a longitudinal evaluation of hyperthyroid patients on treatment, leptin levels were higher and ghrelin levels were lower in the thyrotoxic state than in the euthyroid state. An alternative explanation for the increased appetite needs to be sought in future studies.

## References

[1] Iwen KA, Oelkrug R, Brabant G (2018). Effects of thyroid hormones on thermogenesis and energy partitioning. J Mol Endocrinol.

[2] McAninch EA, Bianco AC (2014). Thyroid hormone signaling in energy homeostasis and energy metabolism. Ann N Y Acad Sci.

[3] Xiao F, Lin M, Huang P, Zeng J, Zeng X, Zhang H (2015). Elevated serum fibroblast growth factor 21 levels in patients with hyperthyroidism. J Clin Endocrinol Metab.

[4] Bande AR, Kalra P, Dharmalingam M, Selvan C, Suryanarayana KM (2019). Serum fibroblast growth factor 21 levels in patients with hyperthyroidism and its association with body fat percentage. Indian J Endocrinol Metab.

[5] Nakamura T, Nagasaka S, Ishikawa S, Hayashi H (2000). Association of hyperthyroidism with serum leptin levels. Metabolism.

[6] Dutta P, Bhansali A, Walia R, Khandelwal N, Das S, Masoodi SR (2012). Weight homeostasis & its modulators in hyperthyroidism before & after treatment with carbimazole. Indian J Med Res.

[7] Oge A, Bayraktar F, Saygili F, Guney E, Demir S (2005). TSH influences serum leptin levels independent of thyroid hormones in hypothyroid and hyperthyroid patients. Endocr J.

[8] Iglesias P, Alvarez Fidalgo P, Codoceo R, Díez JJ (2003). Serum concentrations of adipocytokines in patients with hyperthyroidism and hypothyroidism before and after control of thyroid function. Clin Endocrinol (Oxf).

[9] Kim KJ, Kim BY, Mok JO, Kim CH, Kang SK, Jung CH (2015). Serum concentrations of ghrelin and leptin according to thyroid hormone condition, and their correlations with insulin resistance. Endocrinol Metab (Seoul).

[10] Sadegholvad A, Afkhamizadeh M, Ranjbar-Omrani G (2007). Serum ghrelin changes in thyroid dysfunction. Arch Iran Med.

[11] Riis AL, Hansen TK, Møller N, Weeke J, Jørgensen JO (2003). Hyperthyroidism is associated with suppressed circulating ghrelin levels. J Clin Endocrinol Metab.

[12] ElGawad SS, ElKenawy F, Mousa AA, Omar AA (2012). Plasma levels of resistin and ghrelin before and after treatment in patients with hyperthyroidism. Endocr Pract.

[13] Levy JC, Matthews DR, Hermans MP (1998). Correct homeostasis model assessment (HOMA) evaluation uses the computer program. Diabetes Care.

[14] Cicatiello AG, DiGirolamo D, Dentice M (2018). Metabolic effects of the intracellular regulation of thyroid hormone: old players, new concepts. Front Endocrinol (Lausanne).

[15] Giralt M, Gavaldà-Navarro A, Villarroya F (2015). Fibroblast growth factor-21, energy balance and obesity. Mol Cell Endocrinol.

[16] Mullur R, Liu Y, Brent GA (2014). Thyroid hormone regulation of metabolism. Physiol Rev.

[17] Fisher FM, Kleiner S, Douris N, Fox EC, Mepani RJ, Verdeguer F (2012). FGF21 regulates PGC-1α and browning of white adipose tissues in adaptive thermogenesis. Genes Dev.

[18] Markan KR, Naber MC, Ameka MK, Anderegg MD, Mangelsdorf DJ, Kliewer SA (2014). Circulating FGF21 is liver derived and enhances glucose uptake during refeeding and overfeeding. Diabetes.

[19] Cnop M, Havel PJ, Utzscneider KM, Carr DB, Sinha MK, Boyko EJ (2003). Relationship of adiponectin to body fat distribution, insulin sensitivity and plasma lipoproteins: evidence for independent roles of age and sex. Diabetologia.

[20] Jéquier E (2002). Leptin signaling, adiposity, and energy balance. Ann NY Acad Sci.

[21] Yoshida T, Monkawa T, Hayashi M, Saruta T (1997). Regulation of expression of leptin mRNA and secretion of leptin by thyroid hormone in 3T3-L1 adipocytes. Biochem Biophys Res Commun.

[22] Klok MD, Jakobsdottir S, Drent ML (2007). The role of leptin and ghrelin in the regulation of food intake and body weight in humans: a review. Obes Rev.

